# The Influence of PBF-LB/M Part Forming Angle and Support Structure Parameters on the Distortion of Oral Stent

**DOI:** 10.3390/ma18194588

**Published:** 2025-10-02

**Authors:** Yang Liu, Deqiao Xie, Yihan Liu, Zongjun Tian, Shimao Shangguan, Jinbiao Liao, Zhizhong Hua

**Affiliations:** College of Mechanical and Electrical Engineering, Nanjing University of Aeronautics and Astronautics, Nanjing 210016, China; bravely123@nuaa.edu.cn (Y.L.); dqxie@nuaa.edu.cn (D.X.); liuyihan67@gmail.com (Y.L.); nhsgsm@nuaa.edu.cn (S.S.); liao-jinbiao@nuaa.edu.cn (J.L.); smmhzz@nuaa.edu.cn (Z.H.)

**Keywords:** PBF-LB/M, part forming angles, support structure, stiffness, thin-walled distortion

## Abstract

Powder Bed Fusion-Laser Beam/Metals (PBF-LB/M) enables the layer-by-layer fabrication of complex parts; however, non-uniform thermal transients during the process induce high stresses. Geometric constraints dominate stress–relaxation behavior, which is the primary mechanism leading to part distortion. Therefore, the printing structure serves as a major factor influencing the distortion of PBF-LB/M-fabricated components, of which the forming angle and support structure parameters are the two key factors affecting the printing structure. This study investigates the effects of forming angles and support parameters on the distortion behavior of oral stents manufactured via PBF-LB/M. The results indicate that the magnitude of distortion varies significantly with the forming angle, with the minimum distortion of 0.667 mm occurring at 75°, while the maximum distortion reaches 1.706 mm at 30°. Combined stiffness theory and thermal stress analysis reveal that the thermal stress peaks at a forming angle of 30°, which is governed mainly by the printed cross-sectional area per layer and the cumulative build height. Meanwhile, structural stiffness gradually decreases as the forming angle increases. The study also confirms that support parameters significantly affect distortion, confirming that larger support mesh size and spacing directly contribute to increased maximum distortion. Based on stiffness theory and thermal stress analysis, it is concluded that support structures reduce distortion primarily through two mechanisms: enhancing the overall structural stiffness and facilitating force transmission.

## 1. Introduction

Powder Bed Fusion-Laser Beam/Metals (PBF-LB/M) operates on the principle of layer-by-layer material deposition, with its core advantage lying in the efficient fabrication of complex geometries, including customized components. Given that oral stents require personalized customization based on individual patient anatomy, PBF-LB/M demonstrates substantial potential for application in this field. However, the rapid melt–solidification cycles in PBF-LB/M induce severe heterogeneous thermal stresses, resulting in plastic distortion of parts [1,2,3,4,5]. Compared to existing additive manufacturing technologies (such as Directed Energy Deposition (DED), Binder Jetting (BJ), multi-field assisted printing, multi-material printing, large-scale printing, and hybrid additive–subtractive manufacturing), PBF-LB/M technology offers greater ease of promotion and application, relatively lower cost, and a simpler process flow. Furthermore, it better meets the comprehensive requirements for oral stents in terms of mass production capability, mechanical properties, dimensional accuracy, and surface roughness. Therefore, the PBF-LB/M process was chosen as the research subject for this study [6,7,8].

For oral stents, such distortion has dual detrimental effects: Clinical Impact, as it directly compromises fitting accuracy, significantly reducing patient comfort and potentially causing pain; and Structural Integrity, as it may distort critical structural features, altering force transmission pathways during function. This not only shortens the oral stent’s service life but also jeopardizes the stability of adjacent natural teeth. Consequently, an in-depth investigation into distortion mechanisms and their mitigation strategies is imperative for PBF-LB/M-fabricated oral stents. Forming angles and support structures constitute the primary determinants of distortion from PBF-LB/M use. Accordingly, this work systematically examines the effects of forming angles and support structure parameters on the distortion behavior of oral stents [9,10,11,12].

The forming angle of a part significantly influences its distortion behavior during the PBF-LB/M process. Horizontally built specimens exhibit both lower distortion and reduced residual stress compared to their vertically built counterparts [13]. The average residual stress in horizontal specimens reaches approximately 400 MPa, twice that of samples printed at 90° [14]. Yue Zhou also observed that a 90° build direction yields the best conformity between the as-built and designed contours [15]. Wang et al. demonstrated that when the forming angle exceeds 45°, part distortion decreases significantly, whereas angles below 35° lead to substantially increased distortion [16]. Qiqiang Cao also observed this trend; he compared dimensional accuracy with and without support structures across different forming angles, finding that supported samples showed smaller dimensional errors at 45° and 60°, while unsupported samples performed best at 75°, though the overall accuracy was slightly lower without supports [17].

A number of studies have comprehensively examined the influence of critical support structure parameters, including height, inclination angle, spacing, and type, on the distortion behavior of components fabricated via PBF-LB/M. Zhang et al. demonstrated that an increase in support height reduces the part’s resistance to distortion. Non-inclined support structures exhibited significantly lower warpage distortion compared to inclined supports [18]. The same research team also found that reducing the X/Y offset spacing of supports decreased the magnitude of Z-direction warping distortion, while increasing the support edge offset further contributed to reducing part warpage [19]. The study by S.T. Wu et al. further confirmed that decreasing support spacing effectively minimizes part distortion [20]. A. Dimopoulos compared different types of supports (block, conical, contour, and line) and revealed that line supports minimize cantilever distortion, whereas block supports exhibit the lowest thermal stress [21]. Meanwhile, Khobzi et al. uncovered the correlation between reduced thermal distortion in printed parts and the ability of support structures to alter heat dissipation pathways. The results indicated that increasing the tooth contact area and base conduction area of supports effectively reduces peak temperatures and thermal gradients [22]. Sulaiman et al. verified that decreasing block support spacing and contour support offset reduces distortion while optimizing build time [23]. Structural stiffness enhancement demonstrates distortion resistance; Eric et al. demonstrated that reducing the lattice unit cell size moderately enhances distortion resistance [24]. Tian et al. reduced cantilever distortion through increased lattice wall thickness, attributing this to enhanced structural rigidity [25].

In summary, PBF-LB/M shows strong potential for personalized manufacturing of oral stents. However, part distortion caused by severe non-uniform thermal stresses during fabrication significantly limits its clinical applications. Existing studies indicate that forming angle and support structure parameters considerably influence part distortion. Structural stiffness is also a critical factor affecting distortion. Forming angle and support structure parameters not only regulate the distribution and evolution of thermal stress but also directly determine the structural stiffness of the part. Nevertheless, systematic research on how forming angles and support structures affect distortion through stiffness remains insufficient. Therefore, this study aims to investigate the coupling mechanism between stiffness and thermal stress under various forming angles and support parameters, in order to deepen the understanding of deformation behavior in PBF-LB/M-fabricated oral stents.

## 2. Methods

### 2.1. Experimental Equipment

To address the specific requirements of oral stent fabrication, this study developed a custom metal PBF-LB/M system. The design encompasses seven critical subsystems: optical system, build chamber, powder recoating system, build cylinder, gas circulation system, control system, and frame structure. The developed apparatus exhibits the following features:

(1) A φ180 mm build envelope capable of producing 25 scaffolds per job, equipped with dual lasers;
(2) Maximum scaffold dimensions of 60 mm with build height ≥60 mm;
(3) Bidirectional powder spreading, enhancing production efficiency;
(4) Top-fed powder delivery integrated with a recycling system, improving efficiency while enabling automated production line compatibility;
(5) Optimized gas flow with a main flow zone removing spatter and smoke, and an upper flow zone protecting optical paths while suppressing plume recirculation;
(6) Powder recycling system incorporating cyclonic separation and slag discharge mechanisms to increase powder reuse rate.

This integrated system ensures high-quality printing of oral stents.

The process parameters used in the experiment are shown in Table 1.

**Figure 1:**
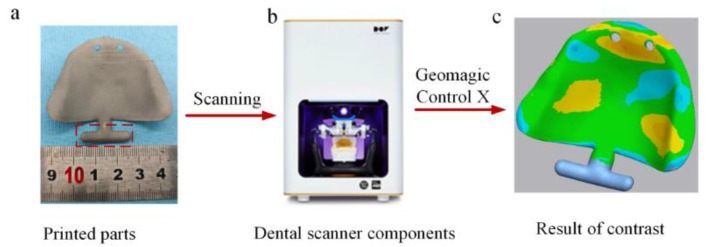
(**a**) Oral stent model. (**b**) DOF Freedom^TM^ UHD scanner. (**c**) Comparison results of the model, before and after printing.

The oral stent model (Figure 1a) was designed using dimensions compliant with removable partial denture specifications according to the industry standard, as per “YY/T 1702-2020: Dentistry—Metal materials for additive manufacturing of fixed and removable dental restorations using laser powder bed fusion” [26]. While accommodating patient-specific anatomical variations, the model was geometrically simplified to facilitate metrological analysis. The area within the red dashed box does not participate in part distortion measurement and is used for scanner fixation.

Point cloud data acquisition was performed using a DOF Freedom™ UHD scanner (DOF Inc., Seoul, Republic of Korea) (Figure 1b), with subsequent export of STL files. Comparative point cloud visualization of the pre- and post-printed oral stent is shown in Figure 1c. The scanned STL files were imported into Geomagic Control X™(Version 2022.1) for best-fit alignment with the original CAD model, enabling quantification of dimensional deviations. Dimensional deviation analysis between as-designed and as-built models was conducted using the best-fit alignment and 3D comparison modules of Geomagic Control X™ software.

**Figure 2:**
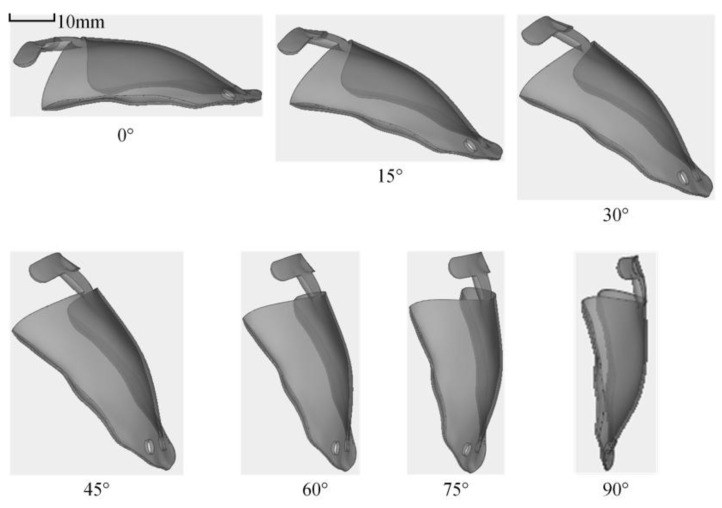
Different forming angles of PBF-LB/M forming parts.

Based on oral stent geometry and clinical considerations, the lingual surface was consistently oriented toward the substrate during fabrication. As depicted in Figure 2, seven distinct forming angles (0°, 15°, 30°, 45°, 60°, 75°, and 90°) were implemented using Voxedance Additive 3.0 software. All other process parameters remained constant, forming angles that constituted the sole variable.

**Figure 3:**
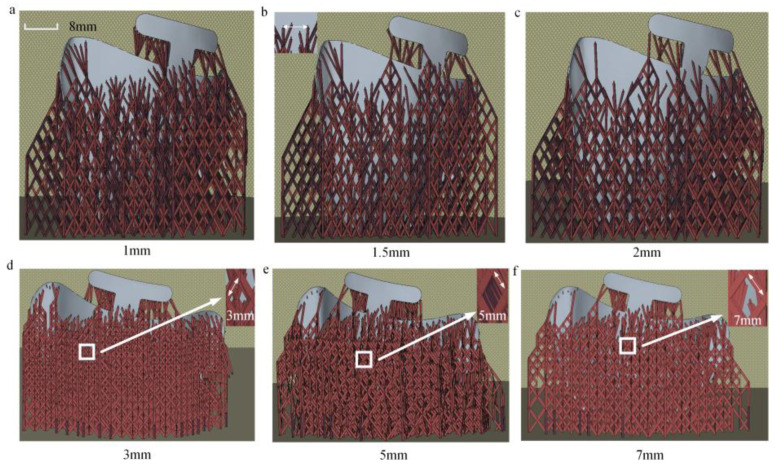
Supporting process parameters. (**a**) The supporting spacing is 1 mm. (**b**) The supporting spacing is 1.5 mm. (**c**) The supporting spacing is 2 mm. (**d**) The mesh size is 3 mm. (**e**) The mesh size is 5 mm. (**f**) The mesh size is 7 mm.

These supports serve not only to secure and position the part but also exhibit varying degrees of thermal conductivity and resistance to distortion, which in turn can significantly influence the distortion of the finished components [18,27,28]. This study investigates the effects of support spacing and support mesh size on the distortion of oral stents through controlled variable experiments. As shown in Table 2, when the forming angle is 75°, the minimum support spacing is 1 mm, while the maximum is 2 mm. The maximum support mesh size is 3 mm, and the minimum is 7 mm. As depicted in Figure 3, the support spacing refers to the distance between the supports on the oral stent, and the support mesh size refers to the length of the rhombus-shaped openings in the support structure.

### 2.2. Simulation Model

#### 2.2.1. Inherent Strain Theory

During PBF-LB/M use on metal components, significant plastic distortion occurs due to the elevated processing temperatures. The inherent strain method incorporating phase transformation effects has been demonstrated as the most accurate simulation approach currently available for additive manufacturing structural analysis. This study utilizes the inherent strain model in Simufact software (Version 4.0) to simulate the distortion and stress of the oral stents. The inherent strain simulation encompasses the sum of elastic strain, plastic strain, thermal strain, and phase transformation strain [29,30,31,32].

Consequently, this study adopts the inherent strain simulation approach. The formula for the strain increments produced in this process primarily includes elastic strain $\varepsilon^{e}$, plastic strain $\varepsilon^{p}$, thermal strain $\varepsilon^{th}$, and phase transformation strain $\varepsilon^{ph}$. Therefore, the total residual strain $\varepsilon_{tot}$ is given by:

(1) $$\varepsilon_{\mathrm{tot}}=\varepsilon^{e}+\varepsilon^{p}+\varepsilon^{th}+\varepsilon^{ph}$$

During the simulation of the mechanical calibration method, the effects of temperature were not explicitly accounted for, and thermal effects were thus neglected. Thermal strain vanishes upon cooling to ambient temperature and is thus excluded from inherent strain analysis [30,31,32,33]. The value of inherent strain is defined as the difference between the total residual strain and the elastic strain, as expressed in Equation (2).

(2) $$\varepsilon^{*}=\varepsilon_{\mathrm{tot}}-\varepsilon^{e}$$

Equation (2) enables the calculation of residual stress through the use of inherent strain. Consequently, the inherent strain constitutes the aggregate of plastic strain, thermal strain, and strain induced by phase transformations, as delineated in Equation (1). Employing the elastic finite element method, the inherent strain is leveraged to determine the residual stress, as articulated in the subsequent Equation (3) [33,34,35,36,37].

(3) $$\left\{\begin{array}{c} \left[K\right]\left[u\right]=\left[f^{*}\right]\\ f^{*}=\int \left[B\right][D][\varepsilon^{*}]dV \end{array}\right.$$

Within this framework, the matrix [*K*] corresponds to the elastic stiffness matrix, the vector [*u*] signifies the nodal displacement vector, and *f* represents the nodal force vector attributable to inherent strains. [D] is the elastic stiffness matrix of the material. Upon solving for the nodal displacement vector [*u*], the total strain ε is ascertained, which subsequently permits the computation of the residual stress σ.

(4) $$\left\{\begin{array}{c} \varepsilon =\left[B\right]\left[u\right]\\ \sigma =[De]([\varepsilon_{\mathrm{tot}}]-[\varepsilon^{*}]) \end{array}\right.$$

where [B] represents the nodal distortion matrix, and [De] denotes the material elasticity matrix.

**Figure 4:**
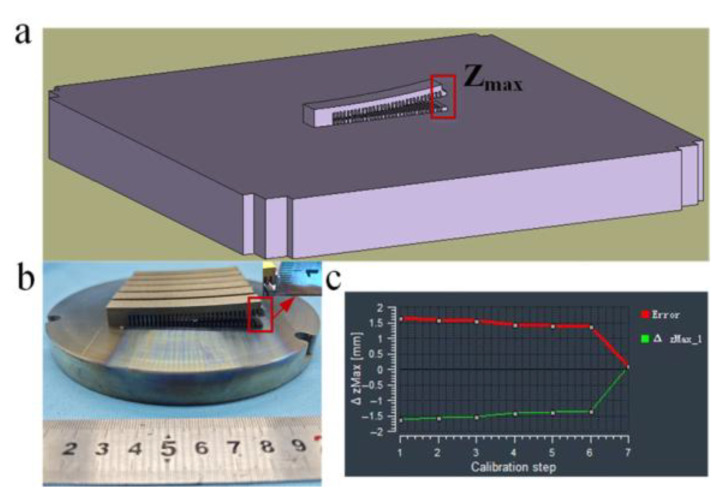
Validation progress. (**a**) Simulated Maximum Distortion. (**b**) Experimental Maximum Distortion. (**c**) Calibration Error.

In this study, the simulated values from Simufact Additive were calibrated. As shown in Figure 4, the simulated and experimental Z_max_ values were compared, and the ΔZ_max_ value was optimized using the least squares method. Six cantilever beam models were printed for this purpose; three were used for calibration, with an experimental average value of 4.211 mm (as shown in Figure 4b), a simulated value of 4.294 mm, and an error of 0.083 mm (as shown in Figure 4c); the other three were used for validation (with laser power values altered compared to the calibration models), yielding an experimental value of 4.62 mm, a simulated value of 4.57 mm, and an error of 0.05 mm.

#### 2.2.2. Simulation Model Setup

**Figure 5:**
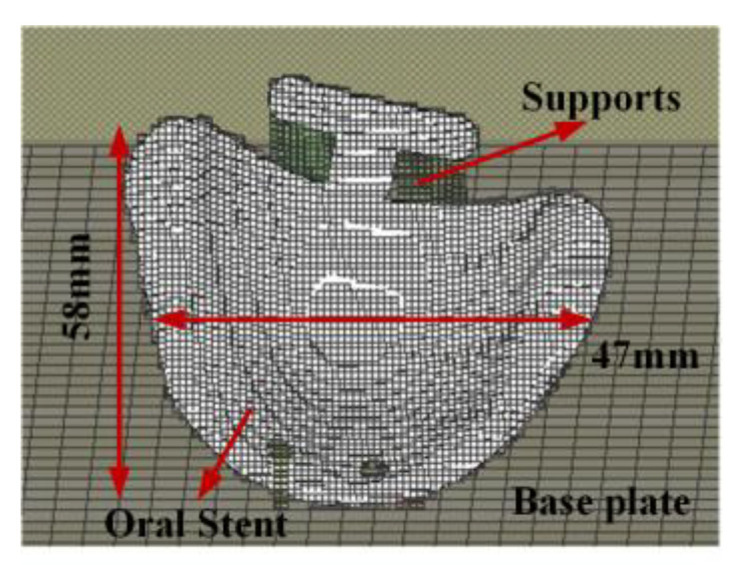
Simulation model of dental support.

As shown in Figure 5, this study used a forming angle of 75° to illustrate the simulation process. The part and support structures were exported separately from the modeling software and then imported together into Simufact Additive. The maximum dimensions of the part were 58 mm × 47 mm, with a mesh size set to 0.37 mm, while the substrate mesh size was set to 3.5 mm. Simufact first calculated the thermal history of the dental scaffold, then determined the inherent strain based on the difference between thermal strain and elastic strain, and finally simulated the forming stress based on the inherent strain. The simulation parameters matched the actual process parameters. To simplify the calculations, the following basic assumptions are proposed [38,39]:

(1) Mechanical assumption: The simulation is treated as a linear elastic static problem, with the material considered isotropic at the macroscopic scale;
(2) Thermal assumption: The complex transient thermo-mechanical coupling process is simplified into an equivalent and calibratable “inherent strain” parameter, which comprehensively reflects the main effects of thermally induced plastic strain;
(3) Geometric assumption: The part, supports, and substrate are all treated as continuous bodies, ignoring the microstructural details of the powder layer.

The thermal conductivity, coefficient of thermal expansion, and specific heat capacity curves for titanium alloy in Simufact are shown in Table 3 and Table 4.

## 3. Results

### 3.1. The Influence of Part Forming Angle on the Distortion of Oral Stents

#### 3.1.1. Summary of Distortion Behavior in Oral Stent Based on Part Forming Angle

This section primarily investigates the distortion of parts at various forming angles ranging from 0° to 90°. It summarizes the distortion patterns under different forming angles and calculates the structural stiffness at each angle.

**Figure 6:**
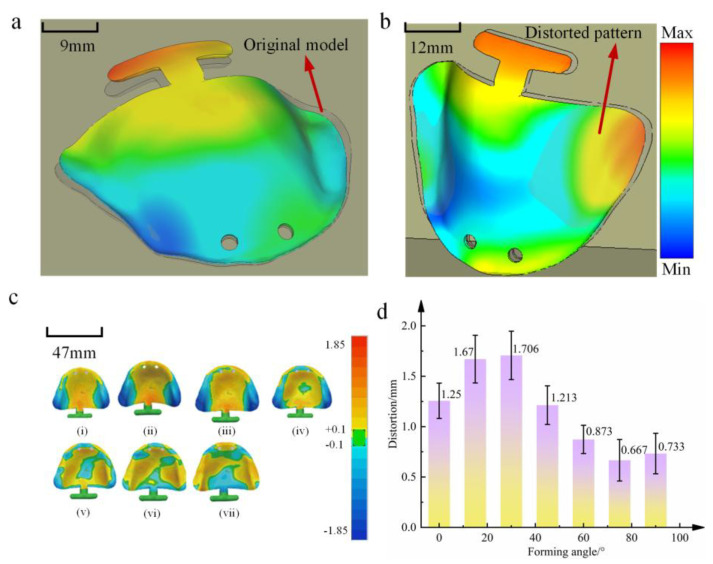
Distortion patterns and distortion laws. (**a**,**b**) Distortion patterns revealed by simulation. (**c**) Distortion patterns observed experimentally ((i) Distortion pattern at 0°, (ii) Distortion pattern at 15°, (iii) Distortion pattern at 30°, (iv) Distortion pattern at 45°, (v) Distortion pattern at 60°, (vi) Distortion pattern at 75°, (vii) Distortion pattern at 90°). (**d**) Distortion laws obtained experimentally.

Figure 6a,b illustrates the two primary distortion modes of the oral stent model. Figure 6a demonstrates mid-span instability with convex distortion induced by axial loading, while Figure 6b exhibits edge-instability-induced warping distortion resulting from fabrication stresses. Figure 6c presents 3D deviation color maps comparing PBF-LB/M -fabricated scaffolds at different forming angles to the original CAD model. Analysis indicates that forming angles significantly affects distortion magnitude. At orientations below 45°, distortion predominantly follows the mode shown in Figure 6a, characterized by substantial mid-scaffold distortion. Conversely, orientations exceeding 45° primarily exhibit the distortion pattern observed in Figure 6b, manifesting as pronounced edge distortion. These are the two deformation modes of flexural instability [43,44,45,46].

As shown in Figure 6d, it can be observed that the distortion of oral stents fabricated by PBF-LB/M is concentrated between 0.69 mm and 1.72 mm. The results are illustrated in Figure 6a. For oral stents fabricated using PBF-LB/M, the distortion ranges from 0.69 mm to 1.72 mm. The distortion of the oral stent model generally increases first and then decreases. When the forming angle is less than 30°, the maximum distortion increases alongside the increase in angle size. However, when the forming angle exceeds 30°, the distortion decreases, showing an overall downward trend. At a forming angle of 75°, the maximum distortion of the part reduces to 0.69 mm, and the overall dimensions become closer to those of the designed model size. When the forming angle is increased to 90°, the maximum distortion of the part is observed to increase slightly, compared to when the angle is 75°.

#### 3.1.2. Analysis of Distortion Mechanisms in Oral Stents Based on Part Forming Angle

**Figure 7:**
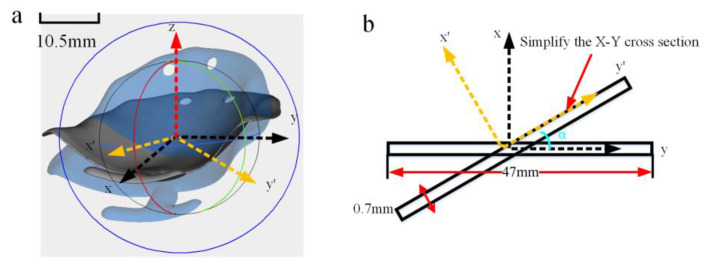
Distortion mechanisms. (**a**) Schematic diagram of stiffness computation for oral stents at varied forming angles. (**b**) Simplified schematic for stiffness computation.

The distortion of oral stents fabricated using PBF-LB/M is influenced by two primary factors: the stresses experienced during the printing process and the structural design of the printed component. Bending stiffness is a crucial parameter for evaluating the resistance of a part to distortion [47]. A higher bending stiffness results in smaller distortion, whereas a lower bending stiffness leads to larger distortion. Therefore, it is essential to investigate the relationship between the bending stiffness and the distortion of oral stents. During the printing process, the distortion of oral stents in the Z-direction is significantly greater than that in the X- and Y-directions. Consequently, this study focuses on the bending stiffness along the Z-axis, as illustrated in Figure 7a,b. To simplify the calculations, the cross-sectional shape of the oral stent in the Y-Z plane is approximated as a rectangle for computational purposes:

(5) $$\left\{\begin{array}{l} K_{1}=EI\\ I=\int_{A}{\left(y\right)}^{2}dA\\ y^{’}=(y\cdot \cos\partial +x\cdot \sin\partial ) \end{array}\right.$$

Solving yields:

(6) $$I=\frac{1}{3}y^{3}{|}_{0}^{7\times 10^{-4}}\cdot x{|}_{0}^{47\times 10^{-3}}\cdot \cos^{2}\partial +\frac{1}{3}x^{3}{|}_{0}^{47\times 10^{-3}}\cdot y{|}_{0}^{7\times 10^{-4}}\cdot \sin^{2}\partial +\frac{1}{4}x^{2}{|}_{0}^{7\times 10^{-4}}\cdot y^{2}{|}_{0}^{47\times 10^{-3}}\cdot \sin2\partial$$

(7) $$I=2.42\times 10^{-8}\cdot \cos^{2}\partial +5.373\times 10^{-12}\cdot \sin^{2}\partial +2.7\times 10^{-12}\cdot \sin2\partial$$

In the formula, *K_1_* represents the bending stiffness of the part, *E* is the elastic modulus of the oral stents, and I is the moment of inertia. The moments of inertia for different part forming angles are presented in Table 5. As the forming angle increases, the bending stiffness of the part gradually decreases. For the supports at different forming angles, their stiffness also diminishes due to the increasing height and decreasing quantity. Consequently, the overall stiffness of the part is reduced. However, the distortion of the part initially increases and then decreases. This indicates that the structural stiffness of the part at different printing angles is not the primary factor influencing the distortion of the part.

**Figure 8:**
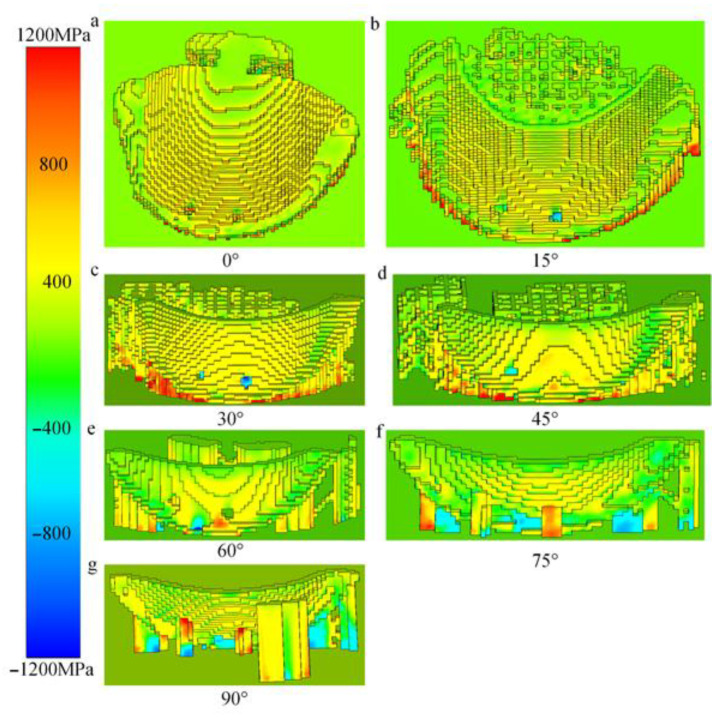
Cross-sectional area and thermal stress at different forming angles.

The primary reason for warping distortion of the part in the Z-direction is the Z-normal stress observed during the simulation. Table 6 shows the distribution of Z-normal stress under different forming angles, indicating that its variation trend aligns with the actual distortion behavior. The differences in distortion behavior among parts with different forming angles mainly stem from dynamic changes in layer height and layer cross-sectional area. As shown in Figure 8a–c, within the forming angle range of 0° to 30°, the cumulative Z-normal stress in the first 20 layers of the part gradually decreases, while the Z-normal stress progressively increases. This is primarily due to the continuous increase in layer height within this angular range. Thus, the distortion behavior in this segment is mainly dominated by the cumulative stress induced by the layer height. In the range of 45° to 75° (Figure 8d–f), the cumulative stress in the first 20 layers also gradually decreases, whereas the Z-normal stress continues to rise. This occurs because the cross-sectional area of the layers within this range progressively decreases, leading to corresponding changes in the distortion mechanism.

### 3.2. Influence of Support Structure Parameters on Distortion of Oral Stents

#### 3.2.1. Summary of Distortion Behavior in Oral Stents Based on Support Structure Parameters

**Figure 9:**
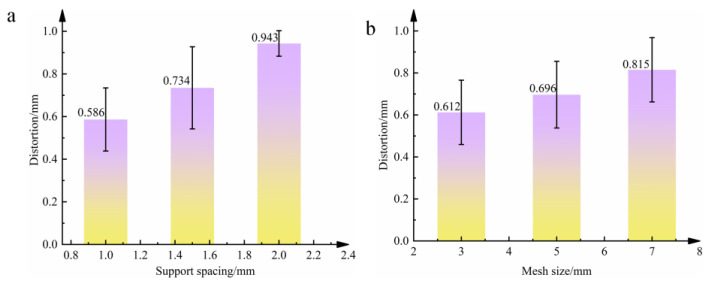
Experimental data. (**a**) Influence of support spacing on the maximum distortion of the support. (**b**) Influence of support mesh size on the maximum distortion of the support.

Figure 9 illustrates the variation in maximum distortion under different support spacing and mesh size conditions. As shown in the figure, when the support spacing increases from 1 mm to 2 mm, the maximum distortion increases from 0.615 mm to 0.895 mm. Similarly, when the mesh size increases from 3 mm to 7 mm, the maximum distortion rises from 0.656 mm to 0.881 mm. The maximum distortion of oral stents is positively correlated with both support spacing and mesh size. In other words, larger support spacing and larger mesh size result in greater part distortion.

#### 3.2.2. Analysis of Distortion Mechanisms in Oral Stents Based on Support Structure Parameters

From Equation (8), it is evident that the greater the cross-sectional area of the supports and the higher the number of supports, the greater the support stiffness, and consequently, the less likely the part is to deform [48]. That is to say, the smaller the support spacing and the smaller the mesh size, the higher the bending stiffness and the smaller the distortion, which is consistent with the experimental results.

(8) $$\left\{\begin{array}{c} I=\frac{b^{4}}{12}\;\\ K=N\cdot E\cdot I \end{array}\right.$$

In Equation (8), *I* represents the moment of inertia of the support cross-section, b denotes the edge length of the support, *K* is the bending stiffness of the support, *N* signifies the number of supports, and *E* is the material’s elastic modulus.

This paper analyzes the influence of the mechanism of support structure parameters on part distortion from a theoretical perspective of bending stiffness. As indicated by Equation (8), larger contact areas between supports and the part, along with a greater number of supports, result in higher overall stiffness of the support structure, thereby reducing the likelihood of component distortion. Consequently, an increase in support spacing or mesh size weakens the stiffness of the support structure, leading to greater distortion. The above theoretical conclusions are consistent with the experimental results presented in this study.

**Figure 10:**
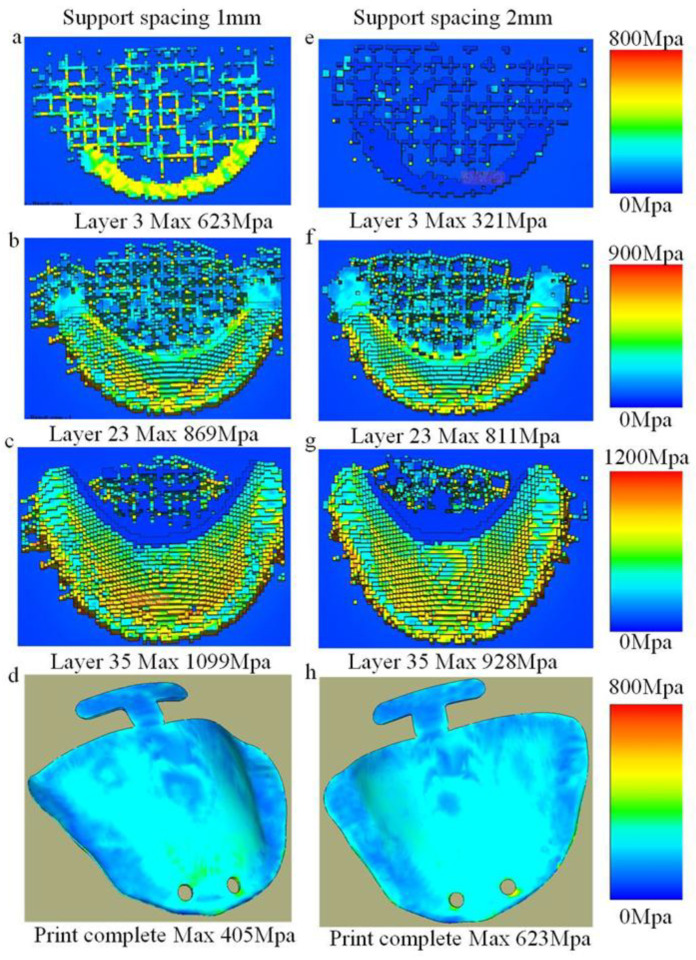
Thermal stress during printing. (**a**–**d**) Support spacing 1 mm printing process. (**e**–**h**) Support spacing 2 mm printing process.

Figure 10 illustrates the thermal stress distributions across different printing layers under support spacings of 1 mm and 2 mm. Subfigures (a) and (e) correspond to the 3rd layer, (b) and (f) to the 23rd layer, (c) and (g) to the 33rd layer, while (d) and (h) depict the thermal stress contour plots after printing completion. During the printing process, the thermal stress under the 1 mm support spacing is significantly higher than that under the 2 mm condition, as evident in subfigures (a), (e), (b), (f), (c), and (g). This increased stress accumulation is attributed to the denser support structure, which imposes stronger mechanical constraints during fabrication. In contrast, after printing is completed, the residual thermal stress with 1 mm support spacing is lower than that with 2 mm spacing, as observed in subfigures (d) and (h). This suggests that the higher support density facilitates more effective stress redistribution and release during cooling.

The pattern of stress variation under different support mesh sizes is consistent with the force transfer mechanism governed by support spacing. During the printing process, a smaller support mesh size leads to higher stress levels in the component; however, after printing is completed, parts with a smaller support mesh size exhibit lower residual stress levels. Therefore, parts with higher support density (smaller support spacing, smaller support mesh size) undergo greater stress during printing, since the larger printed area contributes to increased stress concentration. After printing, however, components with higher support density end up with lower residual stress, due to the greater capacity for stress redistribution and balance provided by the denser support structure. The converse also holds true for all the above relationships.

To conclude, the support serves to enhance the structural rigidity of components and facilitate the transmission of stress.

## 4. Conclusions

This study systematically investigates the influence of printing structural parameters (including part forming angle and support structure parameters) on the distortion behavior of oral stents. The findings reveal the following:

1. Effect of forming angle: As the forming angle increases from 0° to 90°, the maximum distortion magnitude of oral stents exhibits an initial increase followed by a decrease and subsequent increase. The peak distortion occurs at 30° (0.895 mm), while the minimum distortion is observed at 75° (0.615 mm).
2. Distortion pattern by forming angle: When the forming angle is below 45°, distortion predominantly manifests as mid-section warping of the oral stent. Beyond 45°, the distortion pattern notably shifts to peripheral warping.
3. The maximum distortion of oral stents demonstrates a direct proportional relationship with support spacing. Specifically, peak distortion (0.895 mm) occurs at 2 mm spacing. Similarly, distortion magnitude increases proportionally with support mesh size, reaching maximum distortion (0.881 mm) at 7 mm mesh dimensions.

Further mechanistic analysis reveals that:

4. The distortion variation induced by forming angle alterations primarily stems from thermal stress accumulation during printing, exhibiting direct proportionality to both the printed cross-sectional area and printing height. Notably, printing height constitutes the primary contributing factor to distortion at forming angles below 30°, whereas the printed cross-sectional area becomes the dominant influence beyond 30°.
5. Support spacing and mesh size mitigate distortion through enhanced structural rigidity and improved load transfer efficiency. This effect demonstrates an inversely proportional relationship with both parameters, indicating that diminished spacing and smaller mesh dimensions yield progressively more pronounced distortion reduction.

## Figures and Tables

**Table 1:** Process parameters.

Process Parameters	Value
Laser power/W	160
Interlayer corner/°	67
Scanning speed/mm/s	1250
Scanning spacing/mm	0.1
Layer thickness/um	30

**Table 2:** Technical parameters of pure titanium support structure optimization test.

Items	Supporting Generated Critical Angle (°)	Support Spacing (mm)	Support Mesh Size (mm^2^)
1	75	1.0	3
2	75	1.5	5
3	75	2.0	7

**Table 3:** Material properties and processing conditions [40,41,42].

Parameters	Value
Specific heat c (J·Kg^−1^·°C^−1^)	Temperature dependent
Thermal conductivity h (W·m^−1^·°C^−1^)	Temperature dependent
Coefficient of thermal expansion λ (°C^−1^)	Temperature dependent
Ambient temperature (°C)	20
Bulk density ρTA15 (g/cm^3^)	4.450
Radiation emissivity Ɛ	0.36
Poisson’s ratio μ	0.32
Absorptivity Ae	0.7

**Table 4:** Temperature- dependent thermal properties.

T (°C)	Specific Heat (J·kg^−1^·°C^−1^)	Thermal Conductivity (W·m^−1^·°C^−1^)	Coefficient of Thermal Expansion (°C^−1^)
100	545	8.8	8.9
200	587	10.2	9.0
300	628	10.9	9.2
400	670	12.2	9.7
500	712	13.8	10.0
600	755	15.1	10.4
700	838	16.8	10.9
800	880	18.0	10.9
900	922	19.7	10.9

**Table 5:** Product of inertia at different parts-forming angles.

Forming Angle (°)	I (m^4^)
0	2.42 × 10^−8^
15	2.25 × 10^−8^
30	1.8154 × 10^−8^
45	1.2105 × 10^−8^
60	0.605 × 10^−8^
75	0.1475 × 10^−8^
90	5.373 × 10^−12^

**Table 6:** Released stress at different parts-forming angles.

Forming Angle (°)	Released Stress/MPa
0	1416
15	1655
30	2625
45	1065
60	936
75	730
90	820

## Data Availability

The original contributions presented in this study are included in the article. Further inquiries can be directed to the corresponding author.
